# Circulating exosomal hsa_circRNA_0039480 is highly expressed in gestational diabetes mellitus and may be served as a biomarker for early diagnosis of GDM

**DOI:** 10.1186/s12967-021-03195-5

**Published:** 2022-01-03

**Authors:** Bao Jiang, Junfeng Zhang, Xiubin Sun, Chunyan Yang, Guanghui Cheng, Mengru Xu, Siyuan Li, Lina Wang

**Affiliations:** 1 Obstetric Clinic The Second Hospital, Cheeloo College of Medicine, Shandong University, Jinan, 250033 People’s Republic of China; 2 Jinan Maternity and Child Health Care Hospital, Jinan, Shandong China; 3 Department of Biostatistics, School of Public Health, Cheeloo Collage of Medicine, Shandong University, Jinan, 250012 Shandong China; 4 Department of Pediatrics, Liaocheng People’s Hospital, Liaocheng City, 252000 China; 5 Central Research Laboratory, The Second Hospital, Cheeloo College of Medicine, Shandong University, Jinan, 250033 People’s Republic of China; 6 Department of Critical Care Medicine, Peking Union Medical College Hospital, Peking Union Medical College, Chinese Academy of Medical Science, Beijing, China; 7 Center for Reproductive Medicine, Shandong Provincial Hospital Affiliated With Shandong University, Jinan, 250001 China

**Keywords:** Gestational diabetes mellitus, Exosomes, Circular RNA, Diagnostic biomarker, Early pregnancy

## Abstract

**Background:**

Gestational diabetes mellitus (GDM) seriously affects the health of mothers and babies, and there are still no effective early diagnostic markers. Therefore, it is necessary to find diagnostic biomarkers for screening GDM in early pregnancy. Circular RNA (circRNA) is more stable than linear RNA, and can be encapsulated in exosomes and participate in the pathological process of various diseases, which makes it a better candidate biomarker for various diseases. In this study, we attempted to identify the exosomal circRNA biomarkers for detecting early GDM.

**Methods:**

We performed microarray analysis to compare the plasma exosomal circRNA expression profiles of three GDM patients 48 h before and 48 h after delivery. The repeatability of the expression of circRNAs were randomly validated by RT-PCR analysis. Pearson correlation analysis was applied to evaluate the correlation between circRNAs and OGTT level. ROC curve was established to assess the diagnostic value of circRNAs for GDM at different stages.

**Results:**

Plasma exosomal hsa_circRNA_0039480 and hsa_circRNA_0026497 were highly expressed in GDM patients before delivery (*P* < 0.05). The hsa_circRNA_0039480 expression was higher for GDM group than NGT group at different stages, and was also positively correlated with OGTT during the second trimester (*P* < 0.05). The expression of hsa_circRNA_0026497 was higher for GDM group during the third, and second trimesters. And there was a strong correlation between two circRNAs in GDM patients during the first-trimester (r = 0.496, P = 0.014). Hsa_circRNA_0039480 showed significant diagnostic value in the first, second, and third trimesters of pregnancy (AUC = 0.704, P = 0.005; AUC = 0.898, P < 0.001 and AUC = 0.698, P = 0.001, respectively). Notably, the combination of hsa_circRNA_0039480 and hsa_circRNA_0026497 exhibited promising discriminative effect on GDM in the first trimesters (AUC = 0.754, P < 0.001).

**Conclusion:**

Plasma exosomal hsa_cirRNA_0039480 is highly expressed in GDM patients at different stages and may be served as a candidate biomarker for early detection of GDM.

**Supplementary Information:**

The online version contains supplementary material available at 10.1186/s12967-021-03195-5.

## Introduction

Gestational Diabetes Mellitus (GDM) is a metabolic disorder, in which hyperglycemia is developed during pregnancy in women without diabetes. The incidence of GDM is various in different countries and regions, ranging from 1 to 30% of pregnancies, which is highest in Africa, Asia, and India [1]. Epidemiological evidences show a continuous rise of GDM throughout the world [1–3]. The presence of hyperglycaemia during gestation is often associated with various abnormalities, such as obesity, cardiovascular disease, pre-eclampsia and even stillbirth [1]. It is reported that GDM diagnosed at 24–28 weeks of gestation have already affected fetal development [4]. GDM affects maternal and fetal health substantially and it is of necessity to screen the predictive and diagnostic biomarkers for GDM in the early stage of pregnancy [5].

Exosomes belong to a class of small extracellular vesicle (EV) released from various cell types, which play a pathological role in diseases [6]. The emerging potential of exosomes as the source of biomarkers has been explored. circRNAs are a group of non-coding RNAs that characterized by closed loop structure. A plenty of evidences have showed that circRNAs are enriched, conserved and stable in exosomes and are transferred through exosomes to execute function in target cells [7, 8]. Exosomal circRNAs have been supported to be the promising non-invasive diagnostic biomarkers for various diseases, such as cancers and metabolic diseases [9]. A recent microarray study has revealed that there are differential exosomal circRNA expression profiles in unbilical cord blood between GDM patients and controls, implying the significant value of circRNAs in GDM development and fetal growth [10]. Hsa_circRNA_0054633 has been found to be highly expressed in the blood samples of GDM patients and exhibited a potential diagnostic value in the middle and advanced stage of pregnancy [11]. However, little is known about the predictive and diagnostic value of exosomal circRNAs on GDM during the early pregnancy.

Therefore, we performed the microarray analysis to compare the exosomal circRNA profiling in peripheral blood samples of GDM patients and NGTs. The circRNAs with differential expression were mined and validated in the plasma exosomes during different stages of pregnancy. The relevance of exosomal circRNAs of interest with clinical parameters were detected and the diagnostic value on GDM in the early stage were evaluated. We anticipated that the significant findings in our study could shed light on the prediction and diagnosis of early GDM.

## Results

### Participant characteristics

The characteristics of the third-trimester pregnant women and their newborns were illustrated in Table 1. There were no obvious differences with regard to the age, height, delivery gestational age, gestation (G), production (P) of women and neonatal weight in GDM and NGT group (all *P* > 0.05). Notably, significant differences were observed in the prenatal weight, BMI, OGTT test results of mothers between GDM and NGT group (all *P* < 0.05). Parallelly, similar results were obtained by comparing the basic information of women in the first trimester and secondary trimester pregnancy except for the neonatal weight (Additional file 1: Table S2).

**Table 1:** The baseline characteristics of mothers and their neonates for the third-trimester cohort

Variable	GDM (n = 46)	NGT (n = 47)	*P* value
Age (year)	30.15 ± 3.85	28.97 ± 4.04	0.179
Height (m)	1.63 ± 0.04	1.62 ± 0.05	0.475
Weight (kg)	80.77 ± 11.11	73.53 ± 7.84	0.001
BMI	30.50 ± 3.94	28.03 ± 2.55	0.001
Delivery gestational age (day)	270.54 ± 41.81	278.84 ± 7.73	0.623
Gestation (n)	1.72 ± 0.96	1.73 ± 0.90	0.932
Production(n)	1.24 ± 0.43	1.24 ± 0.49	0.860
Systolic pressure (mmHg)	120.78 ± 10.39	121.51 ± 12.87	0.640
Diastolic pressure (mmHg)	77.46 ± 8.21	74.68 ± 8.23	0.068
OGTT 0 h (mmol/L)	5.20 ± 0.65	4.18 ± 0.21	< 0.001
OGTT 1 h (mmol/L)	10.26 ± 1.51	7.65 ± 0.80	< 0.001
OGTT 2 h (mmol/L)	7.65 ± 1.53	6.37 ± 0.80	< 0.001
Neonatal weight (g)	3423.91 ± 426.71	3432.43 ± 359.25	0.923

*BMI* body mass index

### Identification and characterization of exosomes in pregnant women's plasma

**Fig. 1:**
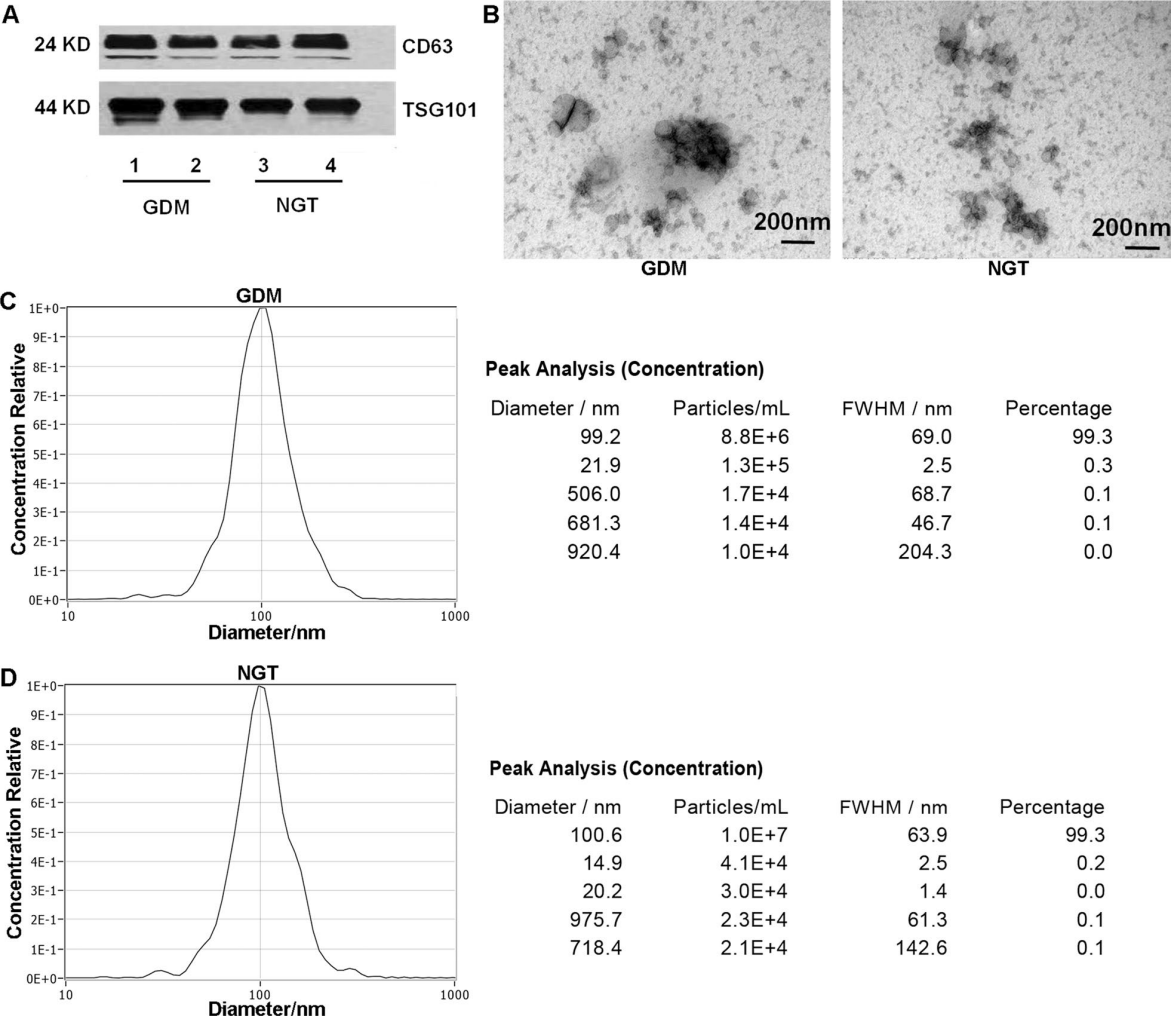
Identification of exosomes isolated from peripheral blood of GDM and NGT pregnancy women. **A** western blot for expression of exosome markers (CD63 and TSG101). The extracted vesicles were positive with CD63 and TSG101 staining. **B** exsomes in GDM and NGT group were observed under electron micrograph. All the vesicles were displayed with typical cup-shaped structure. **C** and **D** the purified particles in GDM and NGT group were analyzed by nanoparticle tracking analysis (NTA). Most of the particles exhibited the size around 100 nm

To identify the exosomes isolated from the peripheral blood of women included in GDM and NGT group, exosomes were labeled with anti-CD63 and anti-TSG101 antibodies. Western blot showed that the vesicles were positive with CD63 and TSG101 staining, which confirmed the presence of exosomes (Fig. 1A). By electron micrograph analysis, vesicles in GDM and NGT group were present with typical cup-shaped structure (Fig. 1B). NTA of the particles size distribution curve indicated a mode of 100 nm (Figure C and D). All these indicated that the extracellular vesicles conformed to the characteristics of exosomes and the plasma exosomes were successfully extracted.

### The expression of circRNAs in plasma exosomes of GDM pregnant women 48 h before and 48 h after delivery

Polymer precipitation method is used to isolate exosomes. This method inevitably co-precipitates freely circulating RNAs and other EVs. Even if the isolates are rich in exosomes, it cannot clearly indicate that circRNAs are derived from exosomes. For the convenience of reading, we abbreviate the circRNAs from exosome-enriched fractions of the plasma samples as exosomal circRNAs.

**Fig. 2:**
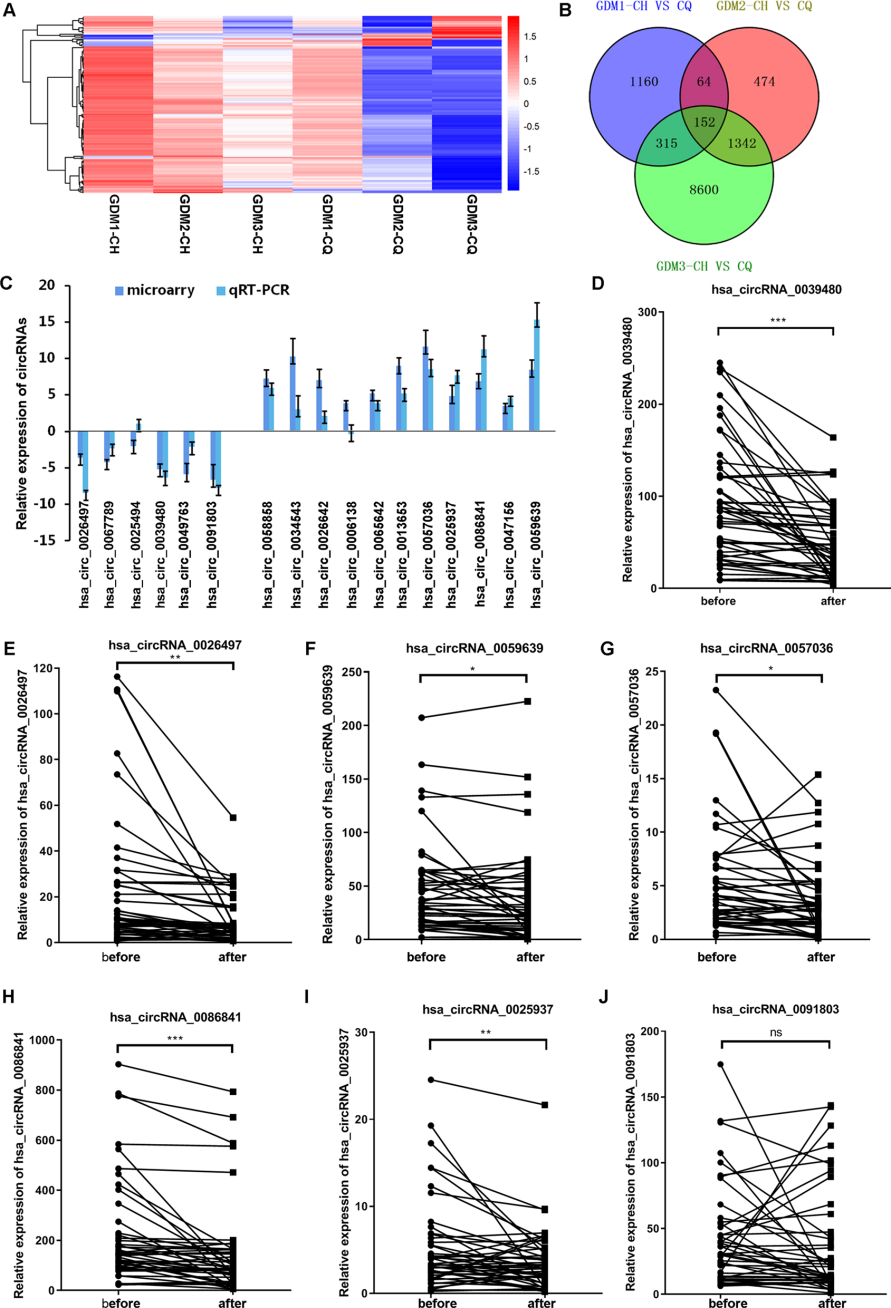
Analysis of differentially expressed exosomal circRNAs. Microarray analysis was performed to screen the differentially expressed exosomal circRNAs in three GDM patients during the third trimester 48 h before delivery, compared with that 48 h after delivery. **A** heat map for the differentially expressed exosome-derived circRNAs in GDM plasma before delivery by hierarchical clustering. The expression profiles of differentially expressed circRNAs could distinguish the GDM exosome samples before delivery and after delivery. **B** the differentially expressed circRNAs were analyzed by Venn diagram analysis. Total 152 overlapped circRNAs were differentially expressed in three paired samples. **C** circRNAs of interest were verified by RT-qPCR analysis in three paired GDM samples. **D**–**J** the repeatability of seven interesting circRNAs were verified by RT-qPCR analysis for the third-trimester GDM cohort. *P < 0.05,**P < 0.01,***P < 0.001, ns, no significant difference

In order to determine the expression profiles of circRNAs in the plasma exosomes of GDM pregnant women, microarray analysis was performed in 3 paired plasma exosome samples 48 h before and 48 h after delivery. As depicted in the heat map, circRNAs were differentially expressed in the plasma exosomes of GDM pregnant patients before delivery, relative to that after delivery (Fig. 2A). According to Venn diagram, there were 152 overlapped circRNAs that differentially expressed in GDM women before delivery, compared with that after delivery, including 92 up-regulated ones and 60 down-regulated ones (Fig. 2B). The expressions of 17 differential circRNAs with highest fold change and smallest p value were screened to be validated by RT-qPCR analysis in the three paired exsomes samples. Figure 2C illustrated that most circRNAs expressions were consistent with the microarray analysis, except for hsa_circ_0025494 and hsa_circ_0006138. Furthermore, the expression of 7 circRNAs was randomly verified by plasma exosome samples from the three-trimester cohort (Fig. 2D–J). Results suggested that the expression of hsa_circRNA_0039480 (Fig. 2D) and hsa_circRNA_0026497 (Fig. 2E) was consistently and significantly higher in GDM patients before delivery than that after delivery (*P* < 0.05), while the remaining ones did not meet expectations. These results revealed a distinct exosomal circRNA expression pattern in GDM patients before and after delivery.

### Expression of hsa_circRNA_0039480 and hsa_circRNA_0026497 in plasma exosomes of GDM/NGT pregnant women before and after delivery

**Fig. 3:**
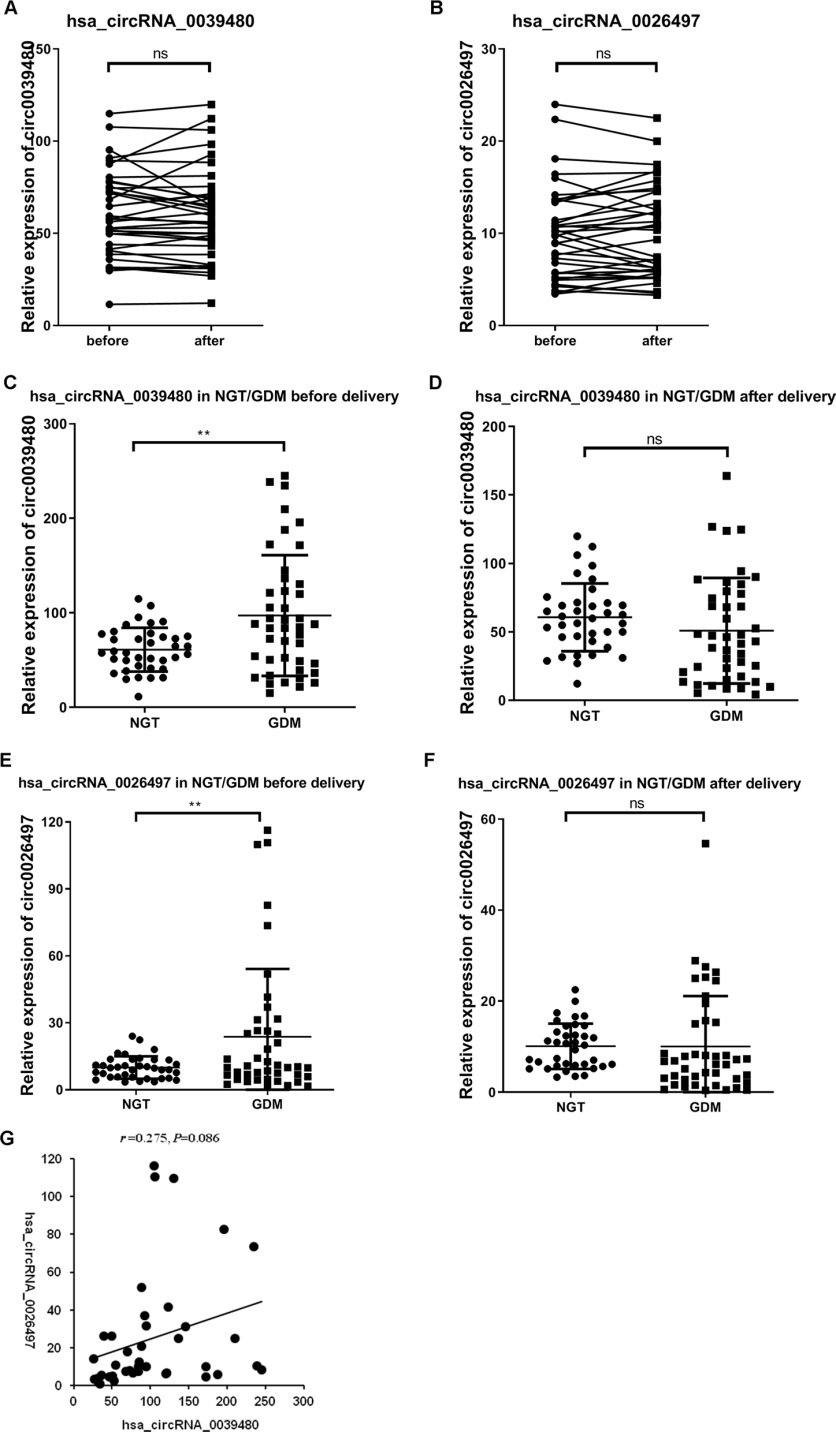
The expression of hsa_circRNA_0039480 and hsa_circRNA_0026497 in plasma exosomes of GDM/NGT pregnancy women before and after delivery. **A** and **B** the expression of hsa_circRNA_0039480 and hsa_circRNA_0026497 in NGT pregnancy women before and after delivery. **C** and **D** comparison of hsa_circRNA_0039480 expression between GDM and NGT group during the third trimester before delivery. **E** and **F** comparison of hsa_circRNA_0026497 expression between GDM and NGT group during the third trimester after delivery. **G** the correlation between hsa_circRNA_0039480 and hsa_circRNA_0026497 expression in GDM patients before delivery by Pearson correlation analysis. **P < 0.01, ns, no significant difference

The two significant circRNAs (hsa_circRNA_0026497 and hsa_circRNA_0039480) screened above were subjected to verification by RT-qPCR for the third-trimester cohort. Results indicated that there was no differential expression of hsa_circRNA_0039480 and hsa_circRNA_0026497 in NGT pregnant women before delivery, compared with that after delivery (*P* > 0.05, Fig. 3A and B). Then, the expressions of hsa_circRNA_0039480 and hsa_circRNA_0026497 in NGT pregnant women were compared with GDM women before and after delivery. Figure 3C and D revealed that the expression of hsa_circRNA_0039480 was significantly higher in GDM group than that in NGT group before delivery (*P* < 0.05), while no significant differences were observed between GDM and NGT group 48 h after delivery (*P* > 0.05). Consistent results were observed in the expression of hsa_circRNA_0026497 for the third-trimester cohort (Figure E and F). In addition, correlation analysis failed to show a strong association in the expression of hsa_circRNA_0039480 and hsa_circRNA_0026497 before delivery (r = 0.275, *P* = 0.086, Fig. 3G).

### Expression of hsa_circRNA_0039480 and hsa_circRNA_0026497 in plasma exosomes of GDM/NGT pregnant women for the first and second cohorts and their correlation with OGTT results during the second trimester

**Fig. 4:**
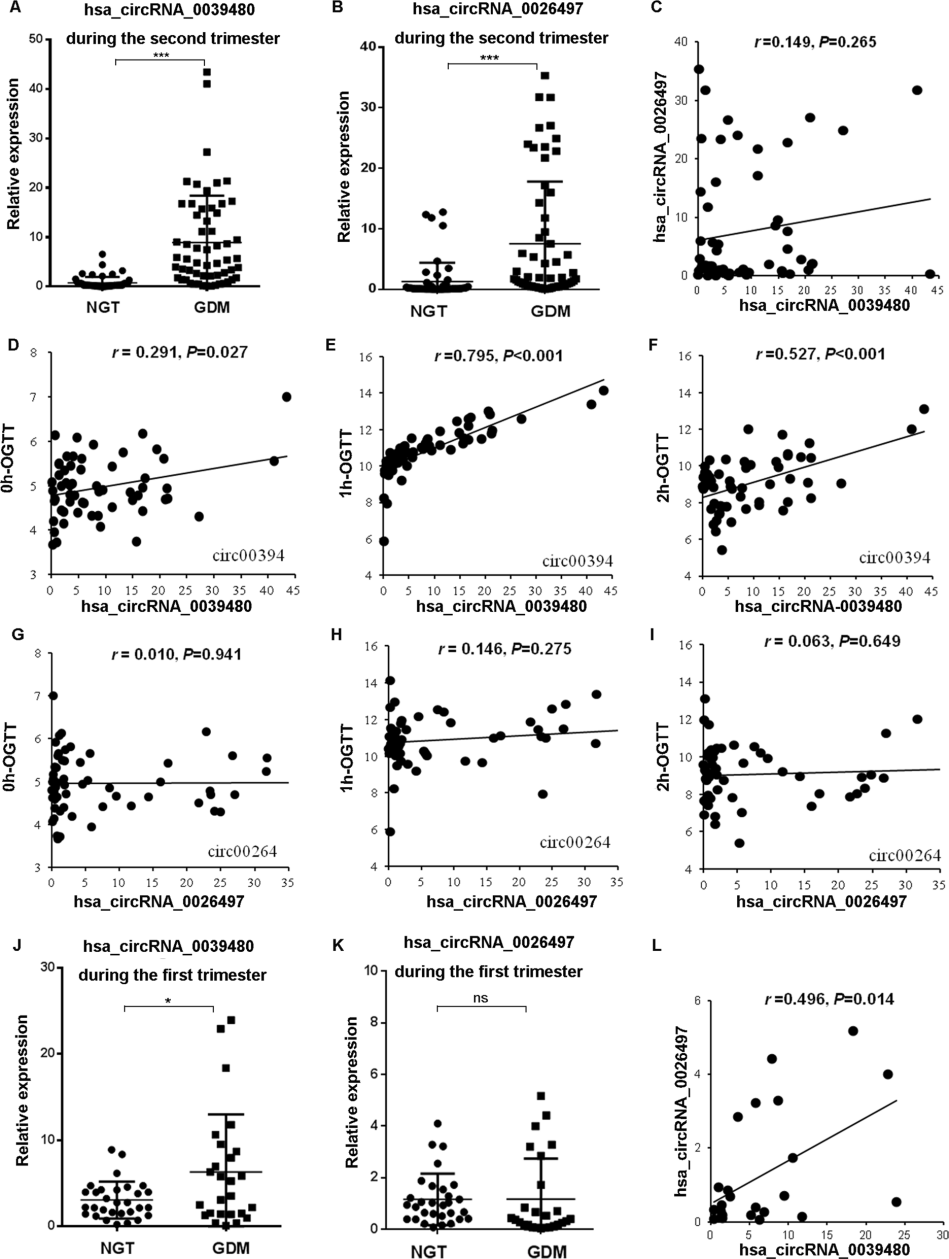
The expression of hsa_circRNA_0039480 and hsa_circRNA_0026497 in plasma exosomes of GDM/NGT pregnancy women during the first and second trimester and their correlation with OGTT glucose level. **A** and **B** the relative expression of hsa_circRNA_0039480 and hsa_circRNA_0026497 in GDM and NGT group for the second-trimester cohort. **C** the correlation between hsa_circRNA_0039480 and hsa_circRNA_0026497 expression in GDM group during the second trimester. **D**–**F** the correlation between hsa_circRNA_0039480 expression and OGTT level at 0, 1 h, and 2 h. **G**–**I** the correlation between hsa_circRNA_0026497 expression and OGTT level at 0, 1 h, and 2 h. **J**, **K** the relative expression of hsa_circRNA_0039480 and hsa_circRNA_0026497 in GDM and NGT group for the first-trimester cohort. **L** the correlation between hsa_circRNA_0039480 and hsa_circRNA_0026497 expression in GDM group during the first trimester. *P < 0.05, ***P < 0.001, ns, no significant difference

The expression profile of hsa_circRNA_0039480 and hsa_circRNA_0026497 during second trimester were first determined by RT-qPCR analysis. As shown in Fig. 4 A and B, the remarkable up-regulation of hsa_circRNA_0039480 and hsa_circRNA_0026497 was found in GDM patients compared with NGT subjects during the second trimester (*P* < 0.05). Nevertheless, the correlation between hsa_circRNA_0039480 and hsa_circRNA_0026497 level during the second trimester was weak (r = 0.149, *P* = 0.265) (Fig. 4C). In order to evaluate the relevance between the expression of hsa_circRNA_0039480 and hsa_circRNA_0026497 with OGTT glucose level, Pearson correlation analysis was performed. As depicted in Fig. 4D–I, there were strong positive correlations between hsa_circRNA_0039480expression and 0 h, 1 h, 2 h-OGTT level (all *P* < 0.05), while no significant relevance was found in hsa_circRNA_0026497 expression with glucose level after OGTT at 0 h, 1 h, and 2 h (all *P* > 0.05). Considering the potential association between circRNAs and glucose level, we further analyzed the expression of hsa_circRNA_0039480 and hsa_circRNA_0026497 in the plasma exosomes for the first-trimester cohort. Results indicated that the expression of hsa_circRNA_0039480 was strikingly increased in GDM group compared with NGT group during the first trimester (*P* < 0.05), while no obvious difference was found in hsa_circRNA_0026497 expression between NGT and GDM group (*P* > 0.05) (Fig. 4J and K). Pearson correlation analysis revealed a positive relationship between hsa_circRNA_0039480 and hsa_circRNA_0026497 expression in the plasma exosomes for the first-trimester GDM cohort (r = 0.496, *P* = 0.014, Fig. 4L). Thus, hsa_circRNA_0039480 was highly expressed in GDM patients during the first and second trimester and its expression was significantly correlated with the OGTT glucose level.

### The diagnostic effect of hsa_circRNA_0039480 and hsa_circRNA_0026497 expression in plasma exosomes on GDM during different stages of pregnancy

**Fig. 5:**
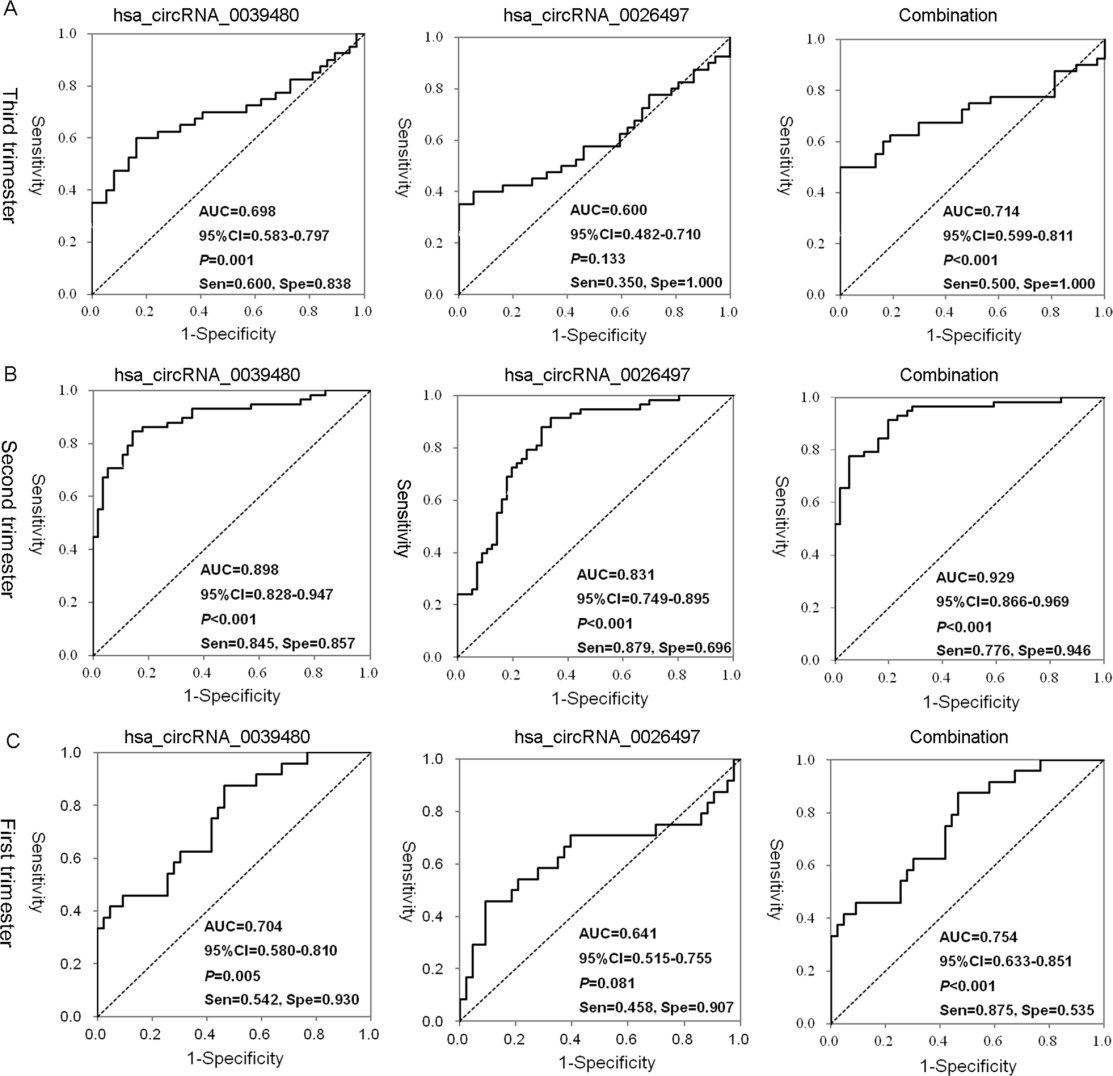
The diagnostic value of hsa_circRNA_0039480 and hsa_circRNA_0026497 for GDM during the first, second, and third pregnancy trimester. ROC curve was established to evaluate the diagnostic performance of hsa_circRNA_0039480 and hsa_circRNA_0026497 for GDM. **A** ROC curves for hsa_circRNA_0039480 and hsa_circRNA_0026497 and their combination during the third trimester. **B**, ROC curves for hsa_circRNA_0039480 and hsa_circRNA_0026497 and their combination during the second trimester. **C** ROC curves for hsa_circRNA_0039480 and hsa_circRNA_0026497 and their combination during the first trimester

To evaluate the diagnostic effect of hsa_circRNA_0039480 and hsa_circRNA_0026497 on GDM, ROC model was established for hsa_circRNA_0039480, hsa_circRNA_0026497 and their combination. Results showed that hsa_circRNA_0039480 expression and hsa_circRNA_0039480−hsa_circRNA_0026497 combination during the third trimester showed a significant diagnostic value in distinguishing GDM and NGT group, but was limited by low sensitivity (≤ 0.600). No significant diagnostic value was observed in hsa_circRNA_0026497 during the third trimester (Fig. 5A). In addition, during the second trimester, a high diagnostic value was achieved for hsa_circRNA_0039480 (AUC = 0.898, sensitivity = 0.845, specificity = 0.857) and hsa_circRNA_0039480-hsa_circRNA_0026497 combination (AUC = 0.929, sensitivity = 0.776, specificity = 0.945). The diagnostic performance of hsa_circRNA_0026497 was limited by relative low specificity of 0.696 (Fig. 5B). Encouragingly, hsa_circRNA_0039480 expression during the first trimester pregnancy exhibited a certain diagnostic value for GDM (AUC = 0.704, sensitivity = 0.542, specificity = 0.930, *P* = 0.005), while hsa_circRNA_0026497 expression did not obtain an idea result with AUC of 0.641, sensitivity of 0.458 and *P* of 0.081. Notably, the combination of hsa_circRNA_0039480 and hsa_circRNA_0026497 showed a promising diagnostic value on GDM during the first trimester (AUC = 0.754, sensitivity = 0.875, specificity = 0.535, *P* < 0.001) (Fig. 5C).

## Discussion

GDM is one of the common metabolic disorders for pregnancy women, which is characterized by elevated blood glucose level in pregnancy and resolved after delivery. GDM has posed the threat to the maternal and fetal health worldwide [12]. Nevertheless, the effectiveness of current management for GDM after diagnosis is limited. In this study, we sought to explore the potential of exosomal circRNAs for the early GDM diagnosis and first identified that exosomal hsa_circRNA_0039480 derived from plasma was a promising diagnostic biomarker for early GDM.

Currently, there are no universally accepted diagnostic protocols or criteria for GDM [12]. At present, the most commonly used diagnostic method for GDM is OGTT at 24–28 weeks of gestation. Previous evidences have indicated that the insulin resistance and blood lipids are obviously increased in GDM patients during 24–28 weeks’ gestation and OGTT has been suggested to be an effective approach for GDM screen [13, 14]. Some researchers argue that GDM has been present in overweight and advanced age women during the early pregnancy (before 20 weeks’ gestation) [9, 15], which have already affected the fetal growth [16]. Another evidence has indicated that there is an association between adverse pregnancy outcomes and the declined blood glucose concentration in GDM patients following 24–28 weeks’ OGTT [17]. Given the potential harm of GDM to mothers and their springs, identification of the diagnostic biomarkers for the early GDM has been an emerging research hotspot. As described in the previous studies, dipocytokine [18], HbA1c [19, 20], glycosylated haemoglobin [21], Adiponectin and 1,5 Anhydroglucitol have been recommended as the biomarker for screening early GDM [22]. However, for the limitation of high cost or labor-intensive procedures, these biomarkers have not been applied in routine use.

CircRNAs belonging to a class of novel discovered non-coding RNAs, have attracted increasing interest in their vital role in various diseases, such as GDM [23]. With high-throughput sequencing technology, circ-0008285 was identified to be highly expressed in plasma samples of GDM patients and might play a pathological role in GDM by regulating total cholesterol and LDL-C levels [24]. Wang et al. reported that circRNAs were deregulated in placentas of GDM patients and predicted to be involved in advanced glycation end products related pathways [25]. In addition, a whole transcriptome analysis of placenta samples from GDM patients showed that there were 114 circRNAs with differential expression, which might affect insulin resistance and lipid metabolism [26]. In this study, we performed microarray analysis to explore the exosomal circRNA expression in the plasma samples of GDM in different stages of pregnancy. Our data showed that hsa_circRNA_0039480 was up-regulated in GDM patients before delivery compared with that after delivery. In line with this, the overexpression of hsa_circRNA_0039480 was determined in GDM patients during the first and second trimester, compared with NGT cohort. All these unraveled the potential role of hsa_circRNA_0039480 in the occurrence and progression of GDM in different stages of pregnancy. Our results also indicated that hsa_circRNA_0039480 expression was positively correlated with OGTT level. We proposed the hypothesis that hsa_circRNA_0039480 might involved in the regulation of blood glucose level. However, the detailed biological role of hsa_circRNA_0039480 in the pathogenesis of GDM deserved further studies.

Besides, a recent evidence revealed that circRNA_0054633 was abnormally expressed in the blood, placenta and cord blood of GDM patients and exhibited a certain value in GDM diagnosis during the second and third trimesters [11]. The differential expressed circulating hsa_circRNA_102893 in the blood samples of GDM patients has been suggested as the potential biomarker for early GDM detection [27]. Recently, exosomes abundant of stable circRNAs have been served as the source of biomarkers [28]. Emerging attentions have been attracted for the potential of exosome derived circulating circRNAs as diagnostic biomarkers in various diseases [29, 30]. However, the predictive role of exosomal circRNAs in early GDM remains to be blank. To our knowledge, it is the first time to unravel the diagnostic role of exosomal circRNAs on GDM in the early pregnancy in our study. Hsa_circRNA_0039480 was overexpressed in GDM patients during different stages of pregnancy and showed a significant diagnostic value on GDM during the first, second and third trimesters of pregnancy (AUC = 0.704, P = 0.005; AUC = 0.898, P < 0.001 and AUC = 0.698, P = 0.001, respectively). Our results were comparable to the previous study of Yang et al., which indicated that hsa_circRNA_102893 could be reliable biomarker for early GDM detection with AUC of 0.741 in ROC test set [27]. Our data also revealed that there was a strong correlation between hsa_circRNA_0039480 and hsa_circRNA_0026497 levels in first-trimester maternal blood (r = 0.496, *P* = 0.014). Encouragingly, the combination of hsa_circRNA_0039480 and hsa_circRNA_0026497 showed excellently discriminative value in distinguishing GDM and NGT group (AUC = 0.754, *P* < 0.001). Since hsa_circRNA_0026497 had limited diagnostic value in the early diagnosis of GDM, combining it did not significantly improve the diagnostic value of hsa_circRNA_0039480. Thus, hsa_circRNA_0039480 may exert function in GDM occurrence by cooperating with hsa_circRNA_0026497 to a certain extent. Further studies about the actual sorting mechanism of exosomal circRNAs are warranted.

In conclusion, plasma-derived exosomal hsa_circRNA_0039480 was highly expressed in different stages of pregnancy. Hsa_circRNA_0039480 showed high predictive value for distinguishing GDM and NGT and could be served as the promising diagnostic biomarker for GDM in the early pregnancy.

Due to the limitation of current methods, the use of polymer precipitation method to isolate exosomes leads to co-precipitation of free circulating RNAs and EVs. Even if the isolate contains abundant exosomes, it cannot clearly indicate that the circRNAs come from exosomes. In any case, whether circRNAs are derived from EVs or free circulating RNAs co-precipitated with EVs, they still have the potential as early markers of gestational diabetes.

## Materials and methods

### Patients and sample collection

This study was a retrospective case–control study and was approved by the Ethics Committee of the Second Hospital of Shandong University. From December 2016 to December 2018, the subjects were enrolled from the Second Hospital of Shandong University. Pregnant women were subjected to a 75-g oral glucose tolerance test (OGTT) during the 24th to 28th weeks of pregnancy. GDM was diagnosed in patients with one or more of the abnormal values, including OGTT fasting ≥ 5.1 mmol/L, 1 h ≥ 10.0 mmol/L and 2 h ≥ 8.5 mmol/L, according to the recommendations of the International Association of Diabetes and Pregnancy Study Groups. Women with other pregnancy-related diseases, such as pre-pregnancy diabetes, chronic hypertension, multiple pregnancy, and pregnancy-induced hypertension were excluded. The GDM patients and age paired normal glucose tolerant (NGT) subjects were included in this study. The blood samples were collected from three cohorts with informed consent, including third trimester (36–41 weeks) group (GDM, n = 46; NGT, n = 47), second trimester (24–26 weeks) group (GDM, n = 58; NGT, n = 56), and first trimester (11–13 weeks) group (GDM, n = 24; NGT, n = 43). The peripheral venous blood specimens were obtained from women 48 h before and 48 h after delivery in third trimester group [31]. The blood samples were collected from pregnant women during Down's screening in the first trimester group and those underwent OGTT in the second trimester, then included them into the GDM and NGT groups based on the results of OGTT. All blood draws used for the experiment were performed under fasting conditions in the morning.

### Exosome isolation

The blood samples (5 mL) were centrifuged at 3,000 rpm for 10 min at room temperature for plasma isolation. Then, the plasma samples were mixed with 0.2 volume of total Exosome Isolation Reagent (Thermo Scientific, Worcester, MA, USA), and incubated at 4 °C for 60 min. Subsequently, exosome-containing pellets were collected by centrifugation at 10,000 rpm for 10 min at 4 °C and suspended in appropriate amount of phosphate buffer saline (PBS). In fact, the use of polymer precipitation to isolate exosomes inevitably co-precipitates freely circulating RNAs and other extracellular vesicles. Therefore, the precipitate is the exosome-enriched fraction of the plasma sample.

### CircRNA microarray

Three GDM patients with mean age of 32.33 ± 6.11 years during the three-trimester pregnancy were included. The basic information of the three patients were listed in Additional file 1: Table S1. Three paired plasma exosome samples extracted from subjects before and after delivery were used for microarray analysis. The circRNA microarray analysis was performed by the Sinotech Genomics Corporation., Shanghai, China. Briefly, the exosome RNA was extracted and purified by using a RNeasy Mini Kit (Qiagen, GmBH, Germany). The integrity and concentration of RNA were checked by an Agilent Bioanalyzer 2100 (Agilent technologies, Santa Clara, CA, US). The qualified RNAs were used for cRNA synthesis and labeling with the application of Sino Human ceRNA array V3.0. Upon hybridization, the array was imaged and scanned by the Agilent Microarray Scanner (Agilent technologies, Santa Clara, CA, US). The raw data were extracted with Feature Extraction software 10.7 (Agilent technologies, Santa Clara, CA, US), and normalized by limma package in R based on Quantile algorithm.

### Reverse transcription (RT) quantitative PCR (qPCR) analysis

Exosome RNA (0.5 μg) was reversely transcribed to cDNA using M-MuL V reverse transcriptase (Fermentas, Hanover, MD, USA), as per the manufacturer’s procedures. The primers for each circRNA were designed by Primer 5 (Table 2). PCR reaction was performed by using the Maxima SYBR Green qPCR Master Mix (Thermo Scientific, Foster City, CA, USA) with the CFX96 Real-Time PCR Detection System (Bio-Rad, Hercules, CA, USA). Conditions for PCR analysis were listed as follows: denaturation at 95 °C for 30 s, followed by 40 cycles of 95 °C for 5 s, 60 °C for 60 s, and 72 °C for 30 s. The qPCR data were analyzed by using 2^−ΔΔCt^ method and normalized to GAPDH. The same procedure was repeated for 3 times.

**Table 2:** Primer sequences for RT-qPCR analysis

circRNAs	Primer sequences for RT-qPCR (5’ to 3’)
hsa_circ_0039480	S: ATGCCCTGAAGAACCGCAG
	AS: TATCCAGAGAGCTTCGACCAG
hsa_circ_0091803	S: ATAGTGCGATGTCGTTTTGTGC
	AS: CAGAATGGACACAGGGAGGTC
hsa_circ_0034543	S: ACCTATGCTGGTGGTAGACT
	AS: CAGCCTATGTGACGAGGGTC
hsa_circ_0067789	S: CCCATCACAATGACACAGAGC
	AS: TCAGGCCGTAAAGTACCTTGG
hsa_circ_0059639	S: AAACCTTTGTGGCTGTGGTTG
	AS: AGTCAAGGAAACACGCTGCC
hsa_circ_0057036	S: AGGTCCCTGCTGAATCACACG
	AS: CTGAGGCGGTGGGGTAAC
hsa_circ_0049861	S: GTGCGGAGGTGTCTGAACTAA
	AS: TTCCTGAGCCCTGTCCAACT
hsa_circ_0086841	S: GCCCCAAACAGGTCATGCT
	AS: GTGTTCTGTGGTTGCCTCCAG
hsa_circ_0049763	S: ACTCATTGTAAGTTGCCACTGC
	AS: TCCAGATGGTTCAACAGCTCA
hsa_circ_0026642	S: GCTAGTCAGTGTGGCTCTCTC
	AS: GGGTCCAGCCAAAGTGATAA
hsa_circ_0065642	S: TGGCACTCAGTCTCTCTTCTCT
	AS: GCCAACTCTACCTGCTTTCCA
hsa_circ_0013653	S: TCAACAGACCGACGTGACAAA
	AS: AACTTTGATGCGTCCTGGCA
hsa_circ_0006138	S: TGCCCGAGTGGTAAAAGACA
	AS: GTCCAATCTGGCTAAAGAGTTGC
hsa_circ_0025937	S: ACAAGCCCGACCAGAATACG
	AS: ACTCCGGGGTTCGAAAATCA
hsa_circ_0026497	S: CAGACAGCTTTGATGACTACCC
	AS: GAAACATAGGTGCTTCCTCCAC

### Western blotting

The exosomal maker proteins of CD63 and Tumor Suspectibility Gene 101 (TSG101) were analyzed by western blot assay. Total protein extraction of the exosome-enriched fraction of the plasma sample was achieved by using the lysis buffer Pro-Prep (iNtRON Biotechnology, South Korea). Proteins (20 μg) were subjected to separation by using a 10% sodium dodecyl sulphate–polyacrylamide gel electrophoresis (SDS-PAGE) system, followed by polyvinylidene fluoride (PVDF) membranes transferring. Subsequently, membranes were incubated with primary antibodies, namely anti-CD63 (1:2,000, ab59479; Abcam), and anti-TSG101 (1:3,000, ab125011; Abcam) and then the secondary antibody of horseradish peroxidase-conjugated anti-rabbit (1:5,000, Novus Biologicals, Littleton, CO, USA). The protein blots were visualized by Western Blotting Luminol Reagent (Bio-Rad, Hercules, CA, USA) incubation and a X-ray film exposure.

### Transmission electron microscopy (TEM)

The exosome-enriched fractions of the plasma samples suspended in PBS were fixed by using 1% glutaraldehyde, and then placed on a copper grid coated with formaldehyde/carbon. Then were stained with 1% uranyl acetate and observed under a JEOL 1200EX electron microscope (JEOL Co., Ltd., Tokyo, Japan).

### Nanoparticle analysis

The size distribution profile of the exosome-enriched fractions of the plasma samples was analyzed by nanoparticle tracking analysis (NTA). The sample at 1 × 10^8^ particles/mL was diluted by PBS and subjected to NTA for 3 cycles at 11 positions. and the particle size and concentration were analyzed by using the built-in ZetaView.

### Statistical analyses

Statistical analysis of the data was performed using the SPSS Statistics 22.0 software (University of Waikato, Hillcrest, NZ). The Shapiro–Wilk test was applied to evaluate the normality of the acquired data. Data with a non-normal distribution was expressed as median and quartile intervals, while the normally distributed data was expressed as mean ± SD. If the data conformed to normal distribution, paired T-test was used to analyze the differences between two groups, otherwise the Wilcoxon test was applied. Pearson’s chi-squared test was used to analyze categorical variables which were represented as numbers and percentages. Correlation between circRNAs and OGTT glucose levels were analyzed by Pearson correlation analysis and ROC curve was constructed to evaluate the diagnostic value of the circRNAs. The significance level for all analyses was set at a p-value of 0.05.

## Supplementary Information

**Additional file 1: Table S1**. The baseline characteristics of mothers and their neonates for the first and second-trimester cohorts. **Table S2**. The detailed clinical information of the three GDM cases for microarray analysis.

## Data Availability

Data can be accessed upon request by e-mailing the corresponding author.

## Abbreviations

circRNAs: Circular RNAs
GDM: Gestational Diabetes Mellitus
EV: Extracellular vesicle
NGT: Normal glucose tolerant
OGTT: Oral glucose tolerance test
ROC: Receiver operating characteristic
TEM: Transmission electron microscopy
AUC: Area Under the Curv

## References

[1] McIntyre HD, Catalano P, Zhang C, Desoye G, Mathiesen ER, Damm P (2019). Gestational diabetes mellitus. Nat Rev Dis Prim.

[2] Chiefari E, Arcidiacono B, Foti D, Brunetti A (2017). Gestational diabetes mellitus: an updated overview. J Endocrinol Invest.

[3] Johns EC, Denison FC, Norman JE, Reynolds RM (2018). Gestational diabetes mellitus: mechanisms, treatment, and complications. Trends Endocrinol Metab.

[4] Kim W, Park SK, Kim YL (2019). Gestational diabetes mellitus diagnosed at 24 to 28 weeks of gestation in older and obese women: is it too late?. PLoS ONE.

[5] Artzi NS, Shilo S, Hadar E (2020). Prediction of gestational diabetes based on nationwide electronic health records. Nat Med.

[6] Kalluri R, LeBleu VS. The biology, function, and biomedical applications of exosomes. Science 2020; 367(6478). 10.1126/science.aau6977 PMC7717626 32029601

[7] Lei M, Zheng G, Ning Q (2020). Translation and functional roles of circular RNAs in human cancer. Mol Cancer.

[8] Ren GL, Zhu J (2019). Noncoding RNAs in acute kidney injury. Kidney Int.

[9] Chen LL (2020). The expanding regulatory mechanisms and cellular functions of circular RNAs. Nat Rev Mol Cell Biol.

[10] Cao M, Zhang L, Lin Y, Li Z, Xu J, Shi Z, Chen Z, Ma J, Wen J (2020). Circular RNA expression profiles in umbilical cord blood exosomes from normal and gestational diabetes mellitus patients. Biosci Rep.

[11] Wu H, Wu S, Zhu Y, Ye M, Shen J, Liu Y, Zhang Y, Bu S (2019). Hsa_circRNA_0054633 is highly expressed in gestational diabetes mellitus and closely related to glycosylation index. Clin Epigenetics.

[12] Szmuilowicz ED, Josefson JL, Metzger BE (2019). Gestational diabetes mellitus. Endocrinol Metab Clin North Am.

[13] Liang Z, Wu Y, Zhu X, Fang Q, Chen D (2016). Insulin resistance and lipid profile during an oral glucose tolerance test in women with and without gestational diabetes mellitus. J Obstet Gynaecol J Inst Obstet Gynaecol.

[14] Zhu WW, Fan L, Yang HX, Kong LY, Su SP, Wang ZL, Hu YL, Zhang MH, Sun LZ, Mi Y, Du XP, Zhang H, Wang YH, Huang YP, Zhong LR, Wu HR, Li N, Wang YF, Kapur A (2013). Fasting plasma glucose at 24–28 weeks to screen for gestational diabetes mellitus: new evidence from China. Diabetes Care.

[15] Zheng W, Xu Q, Huang W, Yan Q, Chen Y, Zhang L, Tian Z, Liu T, Yuan X, Liu C, Luo J, Guo C, Song W, Zhang L, Liang X, Qin H, Li G (2020). Gestational diabetes mellitus is associated with reduced dynamics of gut microbiota during the first half of pregnancy. MSystems.

[16] Ma D, Luque-Fernandez MA, Bogdanet D, Desoye G, Dunne F, Halperin JA (2020). Plasma glycated CD59 Predicts early gestational diabetes and large for gestational age newborns. J Clin Endocrinol Metab.

[17] Yoffe L, Polsky A, Gilam A, Raff C, Mecacci F, Ognibene A, Crispi F, Gratacós E, Kanety H, Mazaki-Tovi S, Shomron N, Hod M (2019). Early diagnosis of gestational diabetes mellitus using circulating microRNAs. Eur J Endocrinol.

[18] Lain KY, Daftary AR, Ness RB, Roberts JM (2008). First trimester adipocytokine concentrations and risk of developing gestational diabetes later in pregnancy. Clin Endocrinol.

[19] International Expert, C (2009). International Expert Committee report on the role of the A1C assay in the diagnosis of diabetes. Diabetes Care.

[20] O'Connor C, O'Shea PM, Owens LA, Carmody L, Avalos G, Nestor L, Lydon K, Dunne F (2011). Trimester-specific reference intervals for haemoglobin A1c (HbA1c) in pregnancy. Clin Chem Lab Med.

[21] Khalafallah A, Phuah E, Al-Barazan AM, Nikakis I, Radford A, Clarkson W, Trevett C, Brain T, Gebski V, Corbould A (2016). Glycosylated haemoglobin for screening and diagnosis of gestational diabetes mellitus. BMJ Open.

[22] Corcoran SM, Achamallah N, Loughlin JO, Stafford P, Dicker P, Malone FD, Breathnach F (2018). First trimester serum biomarkers to predict gestational diabetes in a high-risk cohort: Striving for clinically useful thresholds. Eur J Obstet Gynecol Reprod Biol.

[23] Filardi T, Catanzaro G (2020). Non-coding RNA: role in gestational diabetes pathophysiology and complications. Int J Mol Sci.

[24] Chen H, Zhang S, Wu Y, Li Z, Wang D, Cai S, Wang Z (2021). The role of circular RNA circ_0008285 in gestational diabetes mellitus by regulating the biological functions of trophoblasts. Biol Res.

[25] Wang H, She G, Zhou W, Liu K, Miao J, Yu B (2019). Expression profile of circular RNAs in placentas of women with gestational diabetes mellitus. Endocr J.

[26] Tang L, Li P, Li L (2020). Whole transcriptome expression profiles in placenta samples from women with gestational diabetes mellitus. J Diabetes Investig.

[27] Yang H, Ye W, Chen R, Zeng F, Long Y, Zhang X, Ma J, Gan Q, Rehemutula R, Zhu C (2020). Circulating expression of Hsa_circRNA_102893 contributes to early gestational diabetes mellitus detection. Sci Rep.

[28] Tian Q, He C, Liu G, Zhao Y, Hui L, Mu Y, Tang R (2018). Nanoparticle counting by microscopic digital detection: selective quantitative analysis of exosomes via surface-anchored nucleic acid amplification. Anal Chem.

[29] Pan B, Qin J, Liu X, He B, Wang X, Pan Y, Sun H, Xu T, Xu M, Chen X, Xu X, Zeng K, Sun L, Wang S (2019). Identification of serum exosomal hsa-circ-0004771 as a novel diagnostic biomarker of colorectal cancer. Front Genet.

[30] Wu WP, Pan YH, Cai MY, Cen JM, Chen C, Zheng L, Liu X, Xiong XD (2020). Plasma-derived exosomal circular RNA hsa_circ_0005540 as a novel diagnostic biomarker for coronary artery disease. Dis Markers.

[31] Waters TP, Kim SY, Sharma AJ, Schnellinger P, Bobo JK, Woodruff RT, Cubbins LA, Haghiac M, Minium J, Presley L, Wolfe H, Hauguel-de Mouzon S, Adams W, Catalano PM (2020). Longitudinal changes in glucose metabolism in women with gestational diabetes, from late pregnancy to the postpartum period. Diabetologia.

