# Supplementary material for: Circulating exosomal hsa_circRNA_0039480 is highly expressed in gestational diabetes mellitus and may be served as a biomarker for early diagnosis of GDM
Source: J Transl Med. 2022 Jan 3;20:5. doi: 10.1186/s12967-021-03195-5 (PMC8722188; doi:10.1186/s12967-021-03195-5)
Supplement: Supplementary file 1 — Additional file 1: Table S1. The baseline characteristics of mothers and their neonates for the first and second-trimester cohorts. Table S2. The detailed clinical information of the three GDM cases for microarray analysis. [file 12967_2021_3195_MOESM1_ESM.docx]

Supplemental Table 1 The baseline characteristics of mothers and their neonates for the first and second-trimester cohorts

| Variable | GDM | NGT | P value |
| --- | --- | --- | --- |
| **First trimester** | n=24 | n=43 |  |
| Age (year) | 29.21±3.06 | 28.07±3.73 | 0.207 |
| Height (m) | 1.61±0.05 | 1.62±0.04 | 0.266 |
| Weight (kg) | 76.84±8.23 | 71.10±8.44 | 0.009 |
| BMI | 29.64±2.97 | 27.07±3.03 | 0.001 |
| Delivery gestational age (day) | 272.08±9.82 | 275.79±7.73 | 0.093 |
| Gestation (n) | 2.42±1.41 | 2.07±1.18 | 0.409 |
| Production(n) | 1.58±0.65 | 1.56±0.59 | 0.953 |
| Systolic pressure (mmHg) | 127.50±12.70 | 123.09±8.25 | 0.135 |
| Diastolic pressure (mmHg) | 79.25±6.82 | 78.16±5.91 | 0.497 |
| OGTT 0h (mmol/L) | 5.12±0.44 | 3.94±0.23 | <0.001 |
| OGTT 1h (mmol/L) | 10.13±1.68 | 6.61±1.06 | <0.001 |
| OGTT 2h (mmol/L) | 8.13±1.38 | 6.33±0.97 | <0.001 |
| Neonatal weight（g) | 3455.42±523.15 | 3079.30±327.80 | 0.016 |
| **Second trimester** | n=58 | n=56 |  |
| Age (year) | 32.55±4.43 | 32.05±4.53 | 0.244 |
| Height (m) | 1.62±0.05 | 1.63±0.04 | 0.103 |
| Weight (kg) | 81.35±10.83 | 75.85±6.16 | 0.001 |
| BMI | 31.23±3.65 | 28.46±2.16 | <0.001 |
| Delivery gestational age (day) | 273.76±8.71 | 275.27±7.60 | 0.238 |
| Gestation (n) | 2.31±1.32 | 2.20±1.13 | 0.05 |
| Production(n) | 1.79±0.52 | 1.55±0.54 | 0.018 |
| Systolic pressure (mmHg) | 125.83±8.82 | 123.71±7.45 | 0.166 |
| Diastolic pressure (mmHg) | 81.45±8.46 | 77.84±6.20 | 0.011 |
| OGTT 0h (mmol/L) | 4.96±0.67 | 4.29±0.26 | <0.001 |
| OGTT 1h (mmol/L) | 10.87±1.34 | 7.20±1.14 | <0.001 |
| OGTT 2h (mmol/L) | 9.06±1.50 | 6.34±0.91 | <0.001 |
| Neonatal weight（g) | 3655.00±466.35 | 3055.54±300.79 | <0.001 |

Supplemental Table 2 The detailed clinical information of the three GDM cases for microarray analysis

| Variable | Case 1 | Case 2 | Case 3 |
| --- | --- | --- | --- |
| Age (year) | 39 | 27 | 31 |
| Height (m) | 1.65 | 1.53 | 1.58 |
| Weight (kg) | 85 | 70 | 93 |
| BMI | 31.22 | 29.9 | 37.25 |
| Delivery gestational age (day) | 272 | 285 | 284 |
| Gestation (n) | 3 | 1 | 4 |
| Production(n) | 2 | 1 | 2 |
| Systolic pressure (mmHg) | 123 | 120 | 111 |
| Diastolic pressure (mmHg) | 84 | 90 | 89 |
| OGTT 0h (mmol/L) | 6.52 | 3.99 | 5.83 |
| OGTT 1h (mmol/L) | 14.41 | 12.89 | 10.5 |
| OGTT 2h (mmol/L) | 11.69 | 12.58 | 7.61 |
| Neonatal weight (g) | 3500 | 4400 | 3800 |
